# Pregnancy During the Global COVID-19 Pandemic: Canadian Experiences of Care

**DOI:** 10.3389/fsoc.2021.611324

**Published:** 2021-02-04

**Authors:** Sarah Rudrum

**Affiliations:** Department of Sociology, Acadia University, Wolfville, Canada

**Keywords:** Canada, maternity care, pandemic (COVID-19), midwifery, gender, health

## Abstract

Drawing on journal entries written by a cohort of pregnant Canadians, this article explores how responses to the COVID-19 pandemic shaped access to and experiences of maternity care. Variance in practices among jurisdictions and among provider groups meant that participants had diverse experiences. Nevertheless, I identify clear shared concerns, including fear over giving birth with no familial support, the need for better communications, and challenges entailed when needing to switch providers or travel for care during a state of emergency. Despite a universal health care system, there are gaps and inequities in access to appropriate maternity care in Canada; the pandemic exposed existing access challenges.

## Introduction: Canadian Experiences of Pregnancy Care During the Pandemic

When states of emergency were declared in Canada as a result of COVID-19, many non-essential health services were suspended. Maternity and delivery care of course continued, with changes made to protect patients and to streamline health care toward the pandemic. General practitioners, obstetricians, midwives, and interdisciplinary teams are among the publicly funded primary care options available in Canada. At the best of times, however, access to primary care—as well as to specialist and allied care—varies significantly based on jurisdiction and other factors. In particular, access to midwifery care can be poor outside of urban centers in larger provinces, while in rural areas, access to specialist and sometimes even primary health care can require travel.

To learn about the experiences of pregnant people during the early stages of the pandemic, I recruited individuals who were pregnant in April 2020 to participate in journaling about their care; thus I learned about which practices were helpful or concerning at this time. While jurisdiction and provider type remained important, clear communications and continuity of care were essential: women remarked on the difficulties caused by their absence or were grateful for their presence, identifying *communication* and *continuity* as central to feeling cared for. Good communications practices were idiosyncratic, in that they were not concentrated solely in particular jurisdictions or provider types, though midwives were more frequently cited as providing clear communications. As the pandemic continues, or in the event of another health system crisis, communications and care continuity are areas to prioritize.

## Methodology: Journaling Care

For 10 weeks between April and July 2020, I undertook a mixed-methods, primarily qualitative study in which pregnant Canadians completed journal entries in response to prompts, with at least four entries required for inclusion. At the end of the ten-week period, participants were offered the option to complete any additional weekly entries, responding to a generic prompt, as well as one entry post-delivery, reflecting on their delivery and initial post-partum experiences. Participants completed a demographic survey after consenting to participate and before receiving the first journal prompt. As well as capturing the demography of participants, the survey helped me to reach pregnant individuals across Canada and to recruit a higher number of participants, as I anticipated that over a 10-week period there would be some attrition. In the survey, participants were asked for demographic information such as age, province of residence, weeks pregnant, and other children. Recruitment was via Facebook, with posts to pregnancy and maternity care groups in all provinces and territories. The survey did not include questions about maternity care, which was instead explored over ten-weeks of journal prompts. In the prompts, I asked about experiences of care including changes to care, sources of information, and changing plans. I began with a draft set of prompts, which I revised in response to emerging conditions, including changes to public health protocols and restrictions. I read journal entries each week as they were submitted. At the close of the study, I read all data, first reading all entries by each participant to understand full narratives over time, and then by prompt, in order to begin to identify shared experiences. I identified and grouped themes and for the purposes of this article, have focused extensively on those relating to health care and to a lesser extent social support.

Of the 56 people who completed the survey, 24 went on to complete at least four responses. Among them, six lived in Nova Scotia; four in Ontario; three each in British Columbia and Manitoba; two each in New Brunswick and Prince Edward Island; and one each in Alberta, Newfoundland, Saskatchewan, and Yukon. Their demographic information is included in Table 1, below. Most participants self-identified as white; one was white and First Nations and one was Hispanic and Métis. The percentage of Indigenous people in the study, 8%, was almost double the national percentage: 4.9% of people in Canada are Indigenous (Government of Canada, Statistics Canada, 2017), while Latinx people make up 1.3% of the Canadian population (Government of Canada, Statistics Canada, 2016). Participants ranged in age from 21 to 40 with an average age of 32. One participant indicated their gender as X; all others self-identified as women. There was some diversity of sexuality, with 20 straight participants, three bisexual, and one queer pansexual. Two participants were single, while the rest were married or in common-law relationships. Via postal code classification, I determined whether participants lived in a rural or urban setting. Most participants had one or more children. Ten participants were expecting their first child, among them women who identified that they had previously miscarried. While I worked toward diversity in recruitment, I did not seek for this small study to be representative of the Canadian population. The lack of racialized Canadians—with the exception of the Métis-Hispanic and White-First Nations participants is a limitation of the study, in particular for understanding racialized dimensions of healthcare. However, the diversity among participants in terms of provinces and territories, rural and urban, and intended care providers is a strength of the study. One participant was a second-time surrogate. Pseudonyms, selected by participants and revised if they repeated each other or were actual names, are used throughout. I received ethical approval for the study from Acadia University.

**TABLE 1:** Participant Demographics.

Demographic category	Count	Percent (%)
Ethnicity/race		
European/white	24	92
White and first nations	1	4
Métis and hispanic	1	4
Indigenous (national population: 4.9%)		
Yes	2	8
No	24	92
Gender		
Woman	24	96
X	1	4
Sexuality		
Straight	21	84
Bisexual/pansexual	4	16
Marital Status		
Married/common-law	23	92
Single	2	8
Income (household, annual)		
Less than $25K+	2	8
$25–49K	2	8
$50–74K	2	8
$75–99K	5	20
$100K+	14	56
Urban/rural		
Urban	17	68
Rural	8	32
Expected care provider		
Family Doctor	6	24
Midwife	8	32
Obstetrician	11	44
Other children		
Yes	13	52
No	10	40
No response	2	8
Total (n)	25	

## Background: Access to Universal Care

Across the Canadian provinces and territories, public health responses to COVID-19 have varied, partly due to different courses of disease outbreak but also in relation to their political and economic characteristics. Similarly, while universal health care is national, specific policies and practices surrounding maternity care and particularly midwifery vary significantly among jurisdictions, with differences magnified during the pandemic response. Unregulated and extralegal throughout much of the 20th century, midwifery is now regulated and funded in most of Canada—a process that occurred province-by-province between the mid-1990s and today, and whose reach remains uneven. Midwifery demand tends to outstrip its availability, particularly in rural areas or jurisdictions where regulation is recent and scale-up slow. In most settings, midwives are the only provider type to offer home birth—a difference that gained significance during the pandemic.

As states of emergency were announced in the spring of 2020, some provinces banned home births while others supported the uptick in demand for them. The government of Nova Scotia suspended home births on March 30, despite increased demand and without consulting the midwives’ professional association. While no rationale was reported, it occurred during a time when Nova Scotians were being instructed to stay at home and avoid having guests in the home. The suspension was met by protest and was lifted after a month (Grant, 2020; MacLean, 2020; Sibonney, 2020). Quebec also announced but quickly reversed a homebirth ban, opting instead for specific safety measures for home births (Sibonney, 2020). In Ontario, there was no ban, but some seeking midwifery care were turned away due to lack of capacity (Viau, 2020). The College of Midwives of British Columbia recruited retired and non-practicing midwives to meet demand (Midwives Association of British Columbia, 2020). In most Canadian provinces, there are not enough midwives in ordinary times (Viau 2020); demand for options during the pandemic further exposed the existing shortfall.

Increased interest in home birth, as elsewhere, was a response to two major factors: fear of exposure to the virus in hospitals and concern over restrictions on support people at delivery. All provinces and territories introduced limits to non-medical support people, typically allowing only one. Families were also watching the international context, in which some hospitals, including in New York, demanded that women give birth alone (Caron and Van Syckle, 2020), leading New York’s Governor to issue an executive order on rights during labor (Cuomo and Andrew, 2020; Van Syckle and Caron, 2020) and the World Health Organization to affirm laboring women’s right to a “companion of choice (World Health Organization, 2020b). Newfoundland allowed one support person but required that they leave after the delivery with no in-and-out privileges (Bradbury, 2020; Gillis, 2020). BC’s Center for disease Control stated that one person would be permitted, while BC Women’s Hospital in Vancouver allowed a doula plus one additional support person, stating that doulas are members of the health team (Pole 2020); Yukon hospitals took a similar approach (Yukon Hospitals, 2020). Inconsistency over the role of doulas demonstrates the lack of clarity experienced by pregnant people navigating the emerging policy context during COVID-19. Canada’s three Northern territories had low numbers of COVID-19 cases, likely due to their remote locations and restrictions on gathering and travel (CBC News, 2020; Dawson, 2020; D’entremont et al., 2020), yet nevertheless followed similar guidelines of permitting one support person (Northwest Territories Health and Social Services Authority, 2020; Pearce, 2020; Savikataaq, 2020).

The potential need to travel for emergency or specialist care is an additional concern throughout the Canadian North and in some other rural regions—a prospect made more stressful and costly during the pandemic. Restrictions challenged movements to better support Indigenous births, as protocols often include extended family; however Indigenous advocates worked to create tailored advice (Piapot, 2020). For example, a BC report on cultural safety (First Nations Health Authority and Prinatal Services BC, 2020) acknowledged that the virus was likely to have a disproportionate impact in Indigenous communities, and that health care settings and the wearing of PPE had the potential to retraumatize patients due to the impact of both past disease outbreak and of a painful history with medical personnel and practices in residential schools and other settings. It included specific resources for connecting patients to culturally appropriate resources, such as online smudging or ceremony during social distancing and to a funded Indigenous doula program, as well as more general advice, such as working to build connection despite the need for PPE.

Maternity and neonatal care in Canada remained largely safe from COVID-19 during the study period. A hospital in Alberta had confirmed cases among maternity staff in April 2020 (Rieger, 2020), and a neonatal unit in Vancouver had an outbreak in July 2020 (Daflos, 2020). Such outbreaks, thankfully, remained the exception rather than the norm. For all pregnant individuals, however, the care and support normally available during pregnancy and labor changed, whether through changes to provider type, shifting care online, limiting partners, and/or requiring additional costs or steps.

## Findings and Discussion: Perinatal Challenges

I have organized my data to follow the course of a pregnancy, from prenatal experiences through labor and delivery and into the post-partum period. I discuss primary health care, allied health care, and social supports in each phase. While there are through-lines of concern—including the desire for continuity of care, good communications, and some access to social support—I found that participants characterized each stage according to distinct challenges, discussed below.

### Prenatal Challenges

All provider types in Canada offer comprehensive prenatal care, which monitors health status and facilitates diagnostics. For low-risk pregnancies, the Society of Obstetricians and Gynecologists of Canada recommends care on a schedule with increasing frequency as the due date draws near, with visits every 4 weeks during the first thirty weeks, then moving to every 2 weeks until 37 weeks, at which point a weekly appointment is recommended (Ontario, 2020). Prenatal care experiences varied dramatically among participants, depending on province, timing, and their care needs. Prominent concerns regarding primary care included: the need to switch providers and/or provider-types (due to preference or through necessity as their doctors prioritized emergency work during COVID and reduced their caseloads to protect patients), in-person appointments moving to virtual ones, gaps in care, and traveling for care. As well as for primary care, participants discussed changes in access to allied health care services, which also became harder to access during the pandemic. People’s journal entries recounted experiences with prenatal care that ranged from “frustrating” to “exceptional.”

Continuity of care is a tenet of quality maternity care, as it helps meet medical and psychosocial needs (Perdok et al., 2018; World Health Organization, 2018). However, it is not unusual to switch providers during pregnancy. Midwifery clinics often run waitlists; an opening might precipitate a mid-pregnancy move. A switch to an obstetrician might be necessitated by a newly identified risk factor. Those with no care provider at the outset of pregnancy might move from a temporary to a regular provider. The pandemic increased the potential for changes to care, particularly as in order to avoid exposing patients, some doctors working in multiple settings, including emergency rooms, transferred their pregnant patients to other providers, and at the same time, some patients sought midwifery care to be able to avoid hospital and/or clinical settings (MacLean, 2020; Sibonney, 2020).

Catrina, expecting her first baby in Nova Scotia, was among those who experienced a provider change. She was moved to a new doctor, as hers was working in emergency care and did not want to risk exposing patients. She had since begun to contact midwives in her area because she was worried that she would “be made to do something I hadn’t planned on without proper explanation or without having my options laid out for me.” She found it difficult to contact her care team, which was frustrating. Polly, expecting her second child on Prince Edward Island, also had a change in care, to a nurse-practitioner (NP), as her doctor was similarly “prioritizing in-patients at the hospital.” Primary care NPs tend to work in a collaborative care model with other providers; their training and recruitment has been emphasized to ease doctor shortages and improve access to care (Peckham et al., 2018). Polly was happy with the arrangement, saying, “I’ve had two appointments to date with my NP and I believe I received more thorough care at these two appointments than I ever have. Because most patients were being triaged or seen virtually, but prenatal care was continuing in person, she ... was able to spend over an hour with me at each appointment. I was pleasantly surprised.” However, not all care was available within Catrina’s home province, and, when she later had to later travel to Nova Scotia, she found it stressful, as, unlike during pre-COVID times, she had to complete the round trip in 1 day, in order to be exempt from a 14-days quarantine requirement.

While some patients were required to change providers due to COVID-19, others were hoping for a change. Gwen, a White and First Nations woman expecting her first child in Nova Scotia, did not have a family doctor as is the case with many Canadians. She wrote: “Luckily, I was seeing this OBGYN already for follow up from a surgery ... and she agreed to take me on ... Otherwise, I think I may have had to wait several weeks into my pregnancy before being seen.” While Gwen felt lucky to have any primary care, it was not ideal, as the pandemic affected access. Communicating with her health care team was stressful and frustrating, as for Catrina, and Gwen experienced gaps in healthcare provision. She wrote:

I have had follow-up appointments canceled. Since COVID-19 started, there have been weeks at a time where my OB’s office is closed; they do not have a voicemail at their clinic, so there is no way to get in touch with them. This was very stressful when I was becoming increasingly ill with vomiting and was unable to get help. I am hoping that a midwifery group will take me on at 20 weeks, but as there are no midwives in my area, I would have to travel over an hour to a different “health zone” and I am unsure of the likelihood that they will take me on.

Only three sites offer midwifery care in Nova Scotia, all oversubscribed, so the experience of seeking care far away and being waitlisted is, unfortunately, common. Later, Gwen did get a midwife. She updated her journal, writing:

My midwife presented all options and risks and benefits for every choice I had to make. Everything was explained to me in detail. My midwife was very easily accessible to me via phone or email. I wish I had made the switch from OB to midwife much earlier as the experience was much better. Particularly, communication and allowing me to make informed choices was much better. The care I am receiving from my midwife empowers me, vs. the care from my OB, which was anxiety-provoking due to poor communication and lack of information.

Again, communication stood out as important aspect of quality prenatal care.

Wendy, a bisexual doula who lived in Manitoba and was carrying her first child, also had a midwife—a source of reassurance. She wrote: “I’m not concerned (for myself) about how COVID is impacting hospital policies, because I have been extremely fortunate and got placed with a midwife. I’ve always wanted a home birth anyway—huge dream—so this is about as perfect as perfect gets.” This framing of midwifery access as something to hope for and feel lucky about, expressed by Catrina, Gwen, and Wendy, is a discourse I have observed in previous research (Rudrum and Frank, 2021). Despite the Canada Health Act stating that Canadians must have “reasonable access” to insured services, the positioning of midwifery as a special privilege, unlike other kinds of primary care, reflects the lack of sufficient numbers of midwives in Canada. The responses I gathered suggest that there should be a sufficient number of practicing midwives to serve as primary care providers for all who want their services.

Lila, who identified their gender as X, was a second-time surrogate living in southern Ontario, with a modest household income between $25,000 and $49,000. They would have liked to include midwives in their care, but did not. They explained:

Last pregnancy, I used midwives, but my care was transferred in the last week before I gave birth by C-section (at 36 weeks) due to pre-eclampsia and high blood pressure. Due to those complications, the fertility doctor [...] recommended that I use my OB for [this] entire pregnancy [...] I am bound by legal contract to follow the fertility doctor’s medical advice, so I am using my OB for this pregnancy. I like my OB, but I prefer the care midwives provide.

While Lila could have opted to include midwives in a support role, since they wanted their husband and the intended fathers present during delivery, they chose not to, making a similar calculation about doula care: “the fewer people involved, the better.” As with several others, Lila identified a gap in their care, in their case partly because they were waiting for a COVID-19 test result.

For Maria, expecting her first child in Ontario, a gap in her care meant that a healthy ultrasound came as a great relief, demonstrating that during breaks in care, people were not only waiting but also worrying: “I was also very happy after our 20 weeks ultrasound, since there had been almost 10 weeks since my last appointment. I felt so much relief and joy.” The maximum recommended time between two appointments is 4 weeks, so this was indeed a long break in care. Cjay, a Latina and Métis woman in Manitoba, also found extended periods between appointments stressful, writing: “All these changes have me a little stressed out because there’s more precautions to take when leaving the house but also because there’s less check-ups and appointments.” She found that social supports had also changed, writing: “There is a prenatal nutrition program at the health center where they give you a $40 voucher to spend on healthy foods at the food mart. They used to hand out $10 vouchers every week but they’ve changed it to one for the month so you don’t have to go in as much.” Cjay was in her early 20s and one of two participants listing a low household income (under $25,000); this support was likely important to her family.

Among participants whose appointments were moved to a virtual format, some appreciated the time saved by not having to travel or wait, while others experienced virtual care as akin to a gap in care. Harper, who lived in BC and was planning a homebirth, the same as with her older child, appreciated the extra time that virtual care afforded, but stated, “I miss community, connecting with other pregnant women, feeling connected to practitioners.” Larah, expecting her third child in Saskatchewan, wrote:

This pregnancy has been difficult for me and I could of really used the extra care appointment at around 16 weeks, but our midwives office moved to the WHO’s prenatal schedule and cut out that appointment. I had an appointment around 12 weeks but I felt I didn’t need an in-person visit (the pandemic was new and scary) so I opted for a phone appointment instead. I have not seen my midwife since my initial visit at around 9 weeks pregnant, my next appointment is after my 20 weeks ultrasound in 2–3 weeks. It’s been a long stretch.

Again, the space between appointments exceeded the recommended minimum of an appointment every four weeks during this stage of pregnancy. In particular, Larah was concerned about the potential need for specialist care, and worried that if she needed a fetal echocardiogram (which uses reflected ultrasonic waves to examine the structures and functioning of the heart), as she had during her previous pregnancy, she might have to travel to the nearest major city. This did become necessary, and she described the visit:

The hospital was on lockdown and it was almost eerie how slow everything was. My appointment was 4 h later than it should of been and there was nowhere to go and grab a snack while I waited [...]. I had to sit in a room by myself for the entire time. I had the nurse check on me twice in 4 h, but it was pretty depressing and lonely to have to go through the appointment by myself for that length of time.

The need to travel for care is always disproportionately experienced among people in rural communities, with the pandemic exacerbating its stresses and discomforts. While Larah appreciated being checked on by the nurse during her wait, the length of the wait made those efforts at communication feel insufficient.

Ann, expecting her second child in Nova Scotia, received empathetic and supportive prenatal care from her health team. She wrote:

Despite restrictions and new protocols as a result of COVID, I have felt nothing but supported by the health care system during this pregnancy. On three separate occasions I had concerns with the baby (lack of movement and unexplained pain) .... I called the [care provider] and they encouraged me to not hesitate to come in. I was greeted by a fully masked nurse each time who was nothing but empathetic and helpful ... These visits brought such a peace of mind and I was never made to feel as if they were too busy, or that I was overreacting. They always ended each visit urging me to come back if I ever had any concerns again. My OB was also very supportive.

Ann’s overwhelmingly positive experience of prenatal care, however, stood out as an exception.

As well as changes to primary care, changes in access to allied health care services created worry. Previous infertility or pregnancy loss added to the reliance on care from alternative and allied health professions for some. For example, Margaret, who lived in Yukon and was expecting a first child, wrote of her naturopath that “She was instrumental in identifying hormonal and thyroid problems that were keeping us from getting pregnant, so to have this consistent, positive support essentially cut off at the beginning of a pregnancy was extremely stressful.” Kelly, a single woman in her early 40s living in New Brunswick, was expecting a first child after having experienced a previous miscarriage, and her disappointment about missing acupressure was shared in this context.

Alexandra, expecting her second child in BC, also relied on allied health treatments, and was frustrated to have them canceled, noting the cost of paying insurance for unavailable care:

For almost 3 months now, most pregnancy-related appointments I had scheduled have been cancelled—not even rescheduled or postponed, simply canceled indefinitely. It is incredibly frustrating not being able to go to the chiropractor or acupuncturist to relieve pregnancy pains, let alone prenatal registered massages. Especially when you are paying into insurance for such benefits.

Alexandra is referring to employee benefits, which cover some health services not funded under the Canada Health Act and are typically paid via a combination of employer and employee contributions. Services that are not designated as primary care are typically costly to access in Canada, with fees billed privately or to insurance. The pandemic made such treatments less accessible, even to those with insurance.

### Challenges in Labor

Participants wrote about their plans for delivery, and later, about the delivery itself. Birth location (home vs. hospital) was important to some, though not all, participants. A worry over who would be permitted to be present was prevalent. While all jurisdictions allowed at least one support person, being forced to birth without family support remained a fear.

Harper shared that: “I did a home birth with my first, and I am (more) motivated to do the same with this child, just to stay away from the hospital .... The hospital nearest to me was diverting their maternity cases to another hospital. I’m not sure what the current situation is, but expect to get that information from my midwife closer to my due date.”

Harper’s motivation to stay away from the hospital echoed trends reported in the media, but was not shared by all participants. For example, Kristen, a mother of one living in Manitoba, was flexible:

My birth plan is not terribly specific; I plan to go to the hospital and have a baby. It doesn’t matter to me what happens in between, I just want the baby to be healthy and me to be healthy (in that order). For women who have very specific birth plans, I think they may be feeling more anxious and frustrated with the unknown ... At this point I have accepted that these are exceptional times, and I will do whatever is required to ensure the safety of my family (in terms of public health recommendations).

During her surrogate pregnancy, Lila was particularly worried about who could be present, as her partner and the intended parents all seemed essential. They wrote of hearing about:

... a hospital in Quebec [that] told all pregnant people they had to sign a form agreeing to undergo a c-section or they could not deliver at that hospital. This goes against human rights and is NOT acceptable. I am already worried about who I will be able to have in the delivery room, as I want my husband and BOTH parents to be there, if they want to be.

Lila also worried over whether the intended parents would have to quarantine after arriving from Europe. Cjay too worried about family support at delivery:

Lately I’ve found myself worrying about when it comes time to give birth. The first time I had my boyfriend G. and my mom with me, so I hope they can both be there again, or if I’m only allowed one person this time that’s alright too. I just don’t want to be in there alone. I always think something’s gonna go wrong because I’ve read so many awful stories.

Ann similarly wrote that her biggest worry was that her husband would be absent. Margaret shared this worry:

I am terrified that there is a chance that if things get very bad that my partner will not be allowed at the birth of our child. There is no reason to think this is likely to be the case, especially since there is a lot of pressure to keep one support person with labouring mothers, but it’s always in the corner of my mind, especially when I know so many people are not taking any precautions.

That *no* partner would be permitted seemed unlikely, given that policies throughout Canada allowed at least one person. Nevertheless, this was a prevalent fear, demonstrating how a short-lived poor practice in another jurisdiction, such as New York in the early peak of the pandemic (Caron and Van Syckle 2020), could contribute to fears elsewhere.

Brin was expecting her third child in Alberta. She felt that much of the information around pregnancy and COVID-19 was unclear, and wrote:

My OB has told me that 3 weeks prior to my due date, myself and my immediate family need to self-isolate completely to ensure that no one has COVID and we are able to have a healthy and safe delivery with my husband. This is helpful because it’s a clear guideline. It’s not necessarily easy, but it’s clear, which I appreciate.

Later on, when asked to reflect on what had been difficult or helpful about care during this time, she noted that the requirement felt onerous. She wrote:

I’m nervous about doing a full self-isolation at 36 weeks and having both of our young children at home while I try to finish out my last 3 weeks of work. I feel like this will really make the last portion of the pregnancy extra stressful and I’m just not sure if I’ll be able to work as long as I’d like to, but I understand the risks associated.

Self-isolation 3 weeks before the due date was *not* indicated by public health sources in Alberta or elsewhere, to my knowledge, nor was it mentioned by other participants. It is possible that the information conveyed was a personal preference of the obstetrician or practice, and not based on a public health directive. While participants repeatedly referred to their gratitude for providers who shared information and answered questions, a potential weakness of relying on providers to convey public health advice is that the advice offered might be inconsistent, and, as in this case, might be more onerous to patients than what was recommended by public health directives. Ann had a household income over $100,000, perhaps mitigating the consequences of extended self-isolation, but nevertheless the request felt hard to manage. For many patients, this advice to self-isolate for 3 weeks might be impossible due to work demands, the health care needs of family members, family and household structure or other reasons.

Kristen noticed differences between her previous delivery and birthing during the pandemic; she found these differences to be frustrating at worst, while at best she found that, with fewer people on site, the hospital environment was somewhat calmer than usual:

When we arrived at the hospital, I was in active labor. In addition to all of the standard questions they ask you in triage, I also had to answer COVID screening questions while having contractions. This was very frustrating as I felt it held up the process and delayed my admission. All health care workers wore masks at all times ... Thankfully, I was not required to wear a mask while labouring as I had answered “no” to all of the screening questions. I was the only patient in the shared recovery room, but I am unsure if it just worked out that way or if patients were being deliberately placed in separate rooms.

I was allowed to have two support people, but I chose to just have my husband. I am unsure if there were visitors allowed at the hospital, however my husband’s family and I were in agreement that no one should come to the hospital to see the baby. Therefore, my husband and I were the only ones at the hospital during our stay. This was a marked difference from when my son was born 3 years ago, when we had many visitors and stayed 3 days in the hospital. This time I only stayed overnight. Again, not sure if that was a coincidence or COVID precautions. The hospital itself was very quiet. There was no background din of a busy hallway. There were no people in the hallways. It was a bit strange but also very relaxing. It seemed like there were not any visitors for anyone else, either.

Restrictions on visitors created a calm, if somewhat surreal, birth environment. Lilith, a first-time expectant mother in Ontario, found that other than the lack of nitrous oxide, which she had wanted but been previously advised was not being used due to COVID, delivery care went smoothly. She wrote: “We felt welcomed with our delivery team and did not feel like the care was any different from how it would be normally. Usually our hospital offers a follow-up check in the hospital but unfortunately this is now being offered just by phone, so this likely will not be as thorough, and requires us to then have a hearing test done at a later date that would normally be completed at that follow up.” Most of the changes would come post-partum.

### Challenges in Postpartum Plans

Postpartum care—care for the mother-infant dyad in the 6 weeks after delivery—is an essential part of maternity care throughout Canada, while it varies by jurisdiction and by provider type. Those whose primary care is with a physician may see their doctor once in this period, at the doctor’s office (HealthLinkBC, 2020); in contrast, “quality, continuous care” is built into the midwifery model and, for example, Ontario midwives reported seeing their patients over six times on average in the post-partum period, typically in home visits (Association of Ontario midwives, 2019). When planning for delivery, experienced parents were less worried as a group than first-time parents, yet more worried about loss of medical and social support postpartum. They were able to look back on their previous experiences and the value of the care they received. Feelings about fewer visitors were mixed: here too, some experienced parents remembered that visits can be exhausting as well as supportive. Feelings about support networks also depended on the presence or absence of built-in support, particularly among single mothers. Alongside COVID-19 restrictions, which were easing across Canada at the end of June when the question was asked, concern over a potential “second wave” was prominent. While participants mostly had a manageable post-partum plan, words like “anxiety” and “stress” came up frequently. One way of coping with less access to clinical care was to purchase equipment usually provided in a health care setting such as scales for weighing the baby—a move that depended on a degree of financial security.

For most, postpartum plans included fewer people, though this didn’t necessarily mean feeling less supported. In Nova Scotia, Lynne, who was expecting a second child, welcomed the fact the hospital might not be admitting many visitors. She was anticipating a second wave, and wrote:

I am much more comfortable to have limited visitors at the hospital due to that. Recovery from childbirth is challenging, and being in the hospital with the baby uninterrupted is such a short time, that I don’t necessarily want to have numerous visitors in the hospital this time. I found with my first birth, it prolonged the recovery as there were so many visitors coming to see the baby, which caused unneeded physical stress on me while I am trying to heal from surgery and learn to breastfeed my child.

Wendy also mentioned the potential for a second wave and also recognized the advantage of limits to visitors, writing “our available support network is more than adequate, and ... if anything, finding polite ways to secure time alone with the baby as parents will be the tricky part.” She lived with her parents and near her in-laws, and hoped to have a frank conversation about managing contact in the event of a second wave. With local in-laws and parents in an adjacent province, Margaret wrote: “I worry that [the pandemic] means my parents might not be able to visit for a long time after the baby comes, but my partner’s family will pressure us to spend time with them even if I feel unsafe (I will put my foot down if I need to).” She echoed Wendy in planning ahead for difficult conversations in order to manage contact.

Gwen pointed out the link between uncertain postpartum support and anxiety, particularly for first-time parents, writing:

I am nervous I will not know how to be a good parent. I’ve never been good with kids and haven’t ever enjoyed babysitting. Despite that, I’ve always wanted a child or children of my own. I think that it is normal for a person who is having their first baby to feel nervous. I do think I might feel less nervous if it wasn’t so uncertain what supports will be available to me (thinking support groups, my family, my partners’ family) in the first few weeks of my baby’s life.

Gwen’s isolation added to the worries of a first-time pregnancy.

Among the participants were two single mothers, both expecting their first child. Kelly’s plans hadn’t greatly changed: her mother was certain to be there for the labor and the following week. However, Sinclair, a queer pansexual woman with an income under $25,000, lived in Toronto, a COVID hotspot in the Canadian context (Shah, 2020) and her plan to rely on friends for support was made difficult by the pandemic. She too worried about a second wave:

I don’t even have family that can open their bubble to me so I’m not sure if anyone besides my midwives will even be able to be there. Originally my community was going to come and help me out as a single mother, but that’s a huge risk during this time. I do not feel currently adequately supported and if this continues to when I give birth I definitely won’t feel adequately supported.

Lila had found the post-partum period challenging after their previous surrogacy, and was planning to spend time with their husband and their journal, with a therapist available as needed. In ordinary times, the health care system relies on family, and the precarity of this reliance became apparent during COVID.

Participants anticipated that visits with health care providers might be limited, and as this cohort began to have their babies, this turned out to be the case. Alexandra, who was supported by midwives, spoke for many when she wrote:

I have been mostly concerned with having a lack of postpartum care following being discharged from the hospital. I remember with my first birthing experience, I had the most wonderful and attentive care both in and out of the hospital as there weren’t any crazy protective measures being taken at the time. I received visits from public health nurses regularly to ensure I was well on my way to recovery. However this time, I have been warned there will not be the same sort of care, perhaps only a telephone call once in awhile to check in. So that is certainly a bit concerning! I remember my fears the first time around breastfeeding and the amount of blood I was losing, etc., which was so relieving to be physically examined and reassured that all was well with both baby and I. The support a mother receives postpartum I believe is quite crucial to her emotional, mental and physical well-being and road to recovery.

Others also mentioned potential lack of breastfeeding support as central among post-partum concerns.

Kristen described changes to her post-partum care compared to her previous pregnancy, starting with a public health nurse visit the day after taking her daughter home, which included protocols like mask wearing and sanitizing. She had to monitor her baby’s weight, because, as with her first, she was slow to gain. Kristen described that the places she would have gone to had been closed or allocated to COVID testing. Instead, she describes: “I had to take her to a walk-in clinic to be weighed, which made me nervous as I did not want to bring her around anyone who was sick. I ended up ordering a baby scale online so I would not have to bring her to any clinics or the hospital to be weighed.” Similar to purchasing sonograms, buying a scale was a way to keep care safe at home; the ability to do so relied on having expendable income.

Like other parturient women, these participants were working to balance their needs for privacy, recovery, and connection to the baby with their needs for support and a social network, and doing so in the context of various degrees of isolation due to the pandemic.

Throughout their prenatal, delivery, and post-partum care, participants demonstrated patience and acceptance of changes to care during COVID-19 in their journals about their experiences. Nevertheless, some changes were frustrating and unsettling. Most notably, it was difficult for participants to experience changes to care, gaps in care and poor communication during the pre-natal period and decreased supports in the postpartum period. Prior clinical and social factors shaped how care during COVID was experienced: where shortcomings in care were evident, their impacts were most strongly felt by those with prior pregnancy losses or difficult pregnancies, singles without access to their usual social network during COVID, and to an extent first time parents-to-be.

## Conclusion: COVID Stress and the Importance of Communication, Continuity of Care, and Community Support

While this is an exceptional time to be pregnant, needs during pregnancy have not changed: childbearing participants confirmed that they are still seeking clear, reassuring information, autonomy over where and with whom they seek care and give birth, and social and familial support. This study, which drew on journal entries over a ten-week period, shows that whether or not those needs were met within the pandemic response varied considerably, depending on province, outbreak conditions, provider type, and other factors. Many pressing concerns, including difficulty accessing their provider type of choice, the need to travel for care, and waiting for care, resulted from and highlighted pre-existing shortcomings in the health care system. Some problems, such as the need to change providers or travel for care, were multiplied or heightened by pandemic conditions.

The principles of autonomy, choice, and continuity of care are recognized as contributing to a high standard of maternity care in Canada (Sandall, 1995; McCourt and Stevens, 2006; Vedam et al., 2019), and each of these areas suffered to an extent during the pandemic. Participants approached necessary changes to their health care with flexibility and patience, while continuing to highly value clear communications, continuity of care, and community supports. Some participants mitigated changes to care by purchasing their own quasi-medical equipment such as sonograms or scales. It was when participants experienced long gaps in care, were unable to contact their care team, or were unclear about necessary public health protocols that the stress of pandemic pregnancy was exacerbated. The value placed on communication and the continuous, quality care built into the midwifery model of care, and midwives’ singularity of focus on childbearing, meant that pregnant people seen by midwives experienced fewer major disruptions to care, adding to the case for investment in midwifery care. As pandemic states of emergency continue, or in the event of another health system disruption, it is clear that to support birthing women, communications and continuity of care must be prioritized, and any limits to choice and autonomy of care and location be made cautiously, if at all.

## Data Availability Statement

The original contributions presented in the study are included in the article/Supplementary Material, further inquiries can be directed to the corresponding author.

## Ethics Statement

The studies involving human participants were reviewed and approved by Acadia University Research Ethics Board 2020. The participants provided their written informed consent to participate in this study.

## Author Contributions

The author confirms being the sole contributor of this work and has approved it for publication.

## Funding

This research was supported in part by the Social Sciences and Humanities Research Council.
